# The impact on healthcare, policy and practice from 36 multi-project research programmes: findings from two reviews

**DOI:** 10.1186/s12961-017-0191-y

**Published:** 2017-03-28

**Authors:** Steve Hanney, Trisha Greenhalgh, Amanda Blatch-Jones, Matthew Glover, James Raftery

**Affiliations:** 1 Health Economics Research Group (HERG), Institute of Environment, Health and Societies, Brunel University London, London, UB8 3PH United Kingdom; 2 Nuffield Department of Primary Care Health Sciences, University of Oxford, Oxford, OX2 6GG United Kingdom; 3 Wessex Institute, Faculty of Medicine, University of Southampton, Southampton, SO16 7NS United Kingdom; 4 Primary Care and Population Sciences, Faculty of Medicine, University of Southampton, Southampton General Hospital, Southampton, SO16 6YD United Kingdom

**Keywords:** Research impact, Multi-project programmes, Policy impact, Practice impact, Health gains, Monetisation, Payback Framework, Health technology assessment, World Health Report, Global Observatory

## Abstract

**Background:**

We sought to analyse the impacts found, and the methods used, in a series of assessments of programmes and portfolios of health research consisting of multiple projects.

**Methods:**

We analysed a sample of 36 impact studies of multi-project research programmes, selected from a wider sample of impact studies included in two narrative systematic reviews published in 2007 and 2016. We included impact studies in which the individual projects in a programme had been assessed for wider impact, especially on policy or practice, and where findings had been described in such a way that allowed them to be collated and compared.

**Results:**

Included programmes were highly diverse in terms of location (11 different countries plus two multi-country ones), number of component projects (8 to 178), nature of the programme, research field, mode of funding, time between completion and impact assessment, methods used to assess impact, and level of impact identified.

Thirty-one studies reported on policy impact, 17 on clinician behaviour or informing clinical practice, three on a combined category such as policy and clinician impact, and 12 on wider elements of impact (health gain, patient benefit, improved care or other benefits to the healthcare system). In those multi-programme projects that assessed the respective categories, the percentage of projects that reported some impact was policy 35% (range 5–100%), practice 32% (10–69%), combined category 64% (60–67%), and health gain/health services 27% (6–48%).

Variations in levels of impact achieved partly reflected differences in the types of programme, levels of collaboration with users, and methods and timing of impact assessment. Most commonly, principal investigators were surveyed; some studies involved desk research and some interviews with investigators and/or stakeholders. Most studies used a conceptual framework such as the Payback Framework. One study attempted to assess the monetary value of a research programme’s health gain.

**Conclusion:**

The widespread impact reported for some multi-project programmes, including needs-led and collaborative ones, could potentially be used to promote further research funding. Moves towards greater standardisation of assessment methods could address existing inconsistencies and better inform strategic decisions about research investment; however, unresolved issues about such moves remain.

**Electronic supplementary material:**

The online version of this article (doi:10.1186/s12961-017-0191-y) contains supplementary material, which is available to authorized users.

## Background

The World Health Report 2013 argued that “*adding to the impetus to do more research is a growing body of evidence on the returns on investment*” [1]. While much of the evidence on the benefits of research came originally from high-income countries, interest in producing such evidence is spreading globally, with examples from Bangladesh [2], Brazil [3], Ghana [4] and Iran [5] published in 2015–2016. Studies typically identify the impacts of health research in one or more of categories such as health policy, clinical practice, health outcomes and the healthcare system. Individual research impact assessment studies can provide powerful evidence, but their nature and findings vary greatly [6–9] and ways to combine findings systematically across studies are being sought.

Previous reviews of studies assessing the impact of health research have analysed the methods and frameworks that are being developed and applied [6, 8–13]. An additional question, which has to date received less attention, is what level of impact might be expected from different types of programmes and portfolios of health research.

This paper describes the methods used in two successive comprehensive reviews of research impact studies, by Hanney et al. [6] and Raftery et al. [9], and justifies a sample of those studies for inclusion in the current analysis. We also consider the methodological challenges of seeking to draw comparisons across programmes that go beyond summing the impacts of individual projects within programmes. Importantly, programmes would need to be comparable in certain ways for such cross-programme comparisons to be legitimate.

For this paper, we deliberately sought studies that had assessed the impact of all projects in multi-project programmes, whether coordinated or not. We focused on such multi-project programmes because this approach offered the best opportunities for meaningful comparisons across programmes both of the methods and frameworks most frequently used for impact assessment and, crucially, of the levels of impact achieved and some of the factors associated with such impact. Furthermore, such an approach focused attention on the desirability of finding ways to introduce greater standardisation in research impact assessment. However, we also discuss the severe limitations on how far this analysis can be taken. Finally, we consider the implications of our findings for investment in health research and development and the methodology of research on research impact.

## Methods

The methods used to conduct the two previous reviews on which this study is based [6, 9] are described in Box 1.

**Box 1** Search strategy of two original reviews

**Table:** 

The two narrative systematic reviews of impact assessment studies on which this paper is based were conducted in broadly similar ways that included systematic searching of various databases and a range of additional techniques. Both were funded by the United Kingdom National Institute for Health Research (NIHR) Health Technology Assessment (HTA) Programme.
The searches from the first review, published in 2007, were run from 1990 to July 2005 [6]. The second was a more recent meta-synthesis of studies of research impact covering primary studies published between 2005 and 2014 [9]. The search strategy used in the first review was adapted to take account of new indexing terms and a modified version by Banzi et al. [11] (see Additional file 1: Literature search strategies for the two reviews, for a full description of the search strategies). Although the updated search strategy increased the sensitivity of the search, filters were used to improve the precision and study quality of the results. The electronic databases searched in both studies included: Ovid MEDLINE, MEDLINE(R) In-Process, EMBASE, CINAHL, the Cochrane Library including the Cochrane Methodology Register, Health Technology Assessment Database, the NHS Economic Evaluation Database and Health Management Information Consortium, which includes grey literature such as unpublished papers and reports. The first review included additional databases not included in the updated review: ECONLIT, Web of Knowledge (incorporating Science Citation Index and Social Science Citation Index), National Library of Medicine Gateway Databases and Conference Proceedings Index. In addition to the standard searching of electronic databases, other methods to identify relevant literature were used in both studies. This included in the second review an independent hand-searching of four journals (*Implementation Science*, *International Journal of Technology Assessment in Health Care*, *Research Evaluation*, *Health Research Policy and Systems*), a list of known studies identified by team members, reviewing publication lists identified in major reviews published since 2005, and citation tracking of selected key publications using Google Scholar. The 2007 review highlighted nine separate frameworks and approaches to assessing health research impact and identified 41 studies describing the application of these, or other, approaches. The second review identified over 20 different impact models and frameworks (five of them continuing or building on ones from the first review) and 110 additional studies describing their empirical applications (as single or multiple case studies), although only a handful of frameworks had proven robust and flexible across a range of examples.

For the current study the main inclusion criterion was studies that had attempted to identify projects within multi-project programmes in which investigators had claimed to have made some wider impact, especially on policy or practice, and/or for which there was an external assessment showing such impact. We included only one paper per impact assessment and therefore, for example, excluded papers that reported in detail on a subset of the projects included in a main paper. We did not include studies that reported only on the total number of incidents of impacts on policy claimed for a whole programme, rather than the number of projects claiming to make such impact. We included only those studies where the findings were described in a way that allowed them to be collated with others, then analysed and presented in a broadly standardised way. This meant, for example, that the categories of impacts described by the study had to fit into at least one of a number of broad categories.

We defined the categories as broadly as possible to be inclusive and avoid creating overlapping categories. Following an initial scan of the available studies we identified four impact categories that were broadly compatible with, but not necessarily identical to, the impact categories in the widely-used Payback Framework [14, 15] and the Canadian Academy of Health Sciences adaptation of that framework [10]. The categories were impact on health policy or on a healthcare organisation, informing practice or clinician behaviour, a combined category covering policy and clinician impact, and impact on health gain, patient benefit, improved care or other benefits to the healthcare system.

Studies were included if they had presented findings in one or more of these categories in a way that could allow standardised comparison across programmes. In some cases, the studies presented findings solely in terms of the numbers of projects that had claimed or been shown to have had impact in a particular category. These had to be standardised and presented as percentages. Each study was given the same weight in the analysis, irrespective of the number of individual projects covered by the study. For each of the four categories of impacts we then calculated the median figure for those studies showing the percentage of projects that had claimed to make an impact in that category. We also presented the full range of percentages in each category.

We extracted data on methods and conceptual frameworks for assessment of research impact described in each study, and on categories of factors considered by the authors to be relevant for the level of impact achieved. In identifying the latter, our approach was informed by a range of international research literature, in particular the 1983 analysis by Kogan and Henkel of the importance of researchers and potential users working together in a collaborative approach, the role of research brokers, and the presence of bodies that are ready to receive and use the research findings [16, 17]. Other papers on these and related themes that influenced our approach to the analysis included literature related to North and Central America [18–21], Africa [22], the European Union [23], and the United Kingdom [6, 14, 24], as well as international studies and reviews [25–31].

## Results

Thirty-six studies met the inclusion criteria for this analysis [6, 32–66]. These were highly diverse in terms of the location of the research, nature and size of the funder’s research programme or portfolio, the fields of research and modes of funding, time between completion of the programme and impact assessment, the methods (and sometimes conceptual frameworks) used to assess the impact, and levels of impact achieved. A brief summary of each study is provided in Table 1.

**Table 1:** Thirty-six impact assessment studies: methods, frameworks, findings, factors linked to impact achieved

Author, date, location	Programme/speciality	Methods for assessing health research impact/concepts and techniques	Impacts found	Factors associated with level of impact; comments on methods and use of the findings
Adam et al., 2012 [32]; Catalonia, Spain	Catalan Agency for Health Information, Assessment and Quality – Clinical and health services research	Bibliometric analysis; surveys to researchers (99, 70 responded, 71%); interviews – researchers (15), decision-makers (8); in-depth case study of translation pathways Canadian Academy of Health Sciences framework	Overall, 40 principal investigators (PIs) (of the 70) gave 50 examples of changes; examples included 12 organisational changes of the centre/institution; two public health management; two legal/regulatory (some PIs might have given more than one of these: therefore, total for organisational/management/policy changes: possibly 17–23%, and 20% figure used in this analysis); 29 of the 70 (41%): changed clinical practice	Interactions and participation of healthcare and policy decision-makers in the projects were crucial to achieving impact; the study showed that the Agency achieved the aim of filling a gap in local knowledge needs; study provided useful lessons for informing the funding agency’s subsequent action; the studies “*provide reasons to advocate for oriented research to fill specific knowledge gaps*” ([32], p. 327)
Alberta Heritage Fund for Medical Research, 2003 [33]; Alberta, Canada	Alberta Heritage Fund for Medical Research – Health research	Survey to PIs (100, 50 responded, 50%); interviews with decision makers and users Version of Payback Framework	49% impact on policy; 39% changed behaviour; 40% health sector benefits	Research teams with decision-makers or users more successful than those without
Bodeau-Livinec et al., 2006 [34]; France	French Committee for the Assessment and Dissemination of Technological Innovations (CEDIT) – Health technology assessment (HTA)	Semi-directive interviews with stakeholders affected by the recommendations (14); case studies used surveys in hospitals to examine impact of the recommendations (13) No framework stated, but approach to scoring impact followed earlier studies of the CETS in Quebec reported by Jacob et al. [47, 48]	Widespread interest, “*used as decision-making tools by administrative staff and as a negotiating instrument by doctors in their dealings with management....ten of thirteen recommendations had an impact on the introduction of technology in health establishments*” ([34], p. 161); 7 considerable, 3 moderate: total 77%	Main factor fostering compliance with recommendations “*appears to be a system of regulation*” ([34], p.166) Reviewed other studies: “*All these experiences together with our own work suggest that the impact of HTA on practices and… introduction of new technologies is higher the more circumscribed is the target of the recommendation*” ([34], p. 167)
Brambila et al., 2007 [35]; Guatemala	Population Council – Programme of Operation Research projects in reproductive health in Guatemala	Key informant (KI) interviews; document review; site visits to health centres and non-governmental organisations implementing operational research interventions; scored 22 projects (out of 44 conducted between 1988 and 2001) on indicators: 14 process; 11 impact; 6 context Developed an approach involving process, impact and contextual factors; drew on literature such as Weiss [18] and interactive approaches	Of the 22, 13 projects intervention effective in improving results, three interventions not effective; in 14 studies implementing agency acted on results; nine interventions scaled up in same organisation; five adopted by another organisation in Guatemala; some studies led to policy changes, mainly at the programme level (total 64% impact in combined policy and practice category)	Highlighted how impact can arise from a long-term approach and the several 5-year cycles of funding “*allowed for the accumulation of evidence in addition to the development of collaborative ties between researchers and practitioners, which ultimately resulted in changes to the service delivery environments*” ([35], p. 242)
Buxton et al., 1999 [36]; United Kingdom	NHS North Thames Region – Wide-ranging responsive mode R&D programme	Questionnaires to PIs (164, 115 responded, 70%) and some bibliometric analysis for all projects and case studies (19); case studies included interviews with researchers and some users Benefit scoring system based on two criteria (importance of the research to the changes, and level at which the change was made) was used to score questionnaire responses about the impacts and re-score the impact from each study on which a case study conducted Payback Framework	41% impact on policy; 43% change in practitioner/manager behaviour; 37% led to benefits to health and health service	The survey/case study comparison suggests “*greater detail and depth of the case studies often leads to a somewhat different judgement of payback, but there is no evidence of a systematic under-assessment of payback from the questionnaire approach, nor, generally, of greatly exaggerated claims being made by researchers in the self-completed questionnaires*” ([36], p. 196)
Caddell et al., 2010 [37]; Canada	IWK Health Centre, Halifax, Canada, Research Operating Grants (small grants) – Women and children’s health	Online questionnaire to PIs and co-investigators (Co-Is) (64, 39 responded, 61%) Research Impact Framework: adapted	16% policy impact: 8% in health centre, 8% beyond; 32% said resulted in a change in clinical practice; 55% informed clinical practice by providing broader clinical understanding and increased awareness (average of 43% for practice impact); 46% improved quality of care	An association between presenting at conferences and practice impacts; authors stress link between research and excellence in healthcare: “*It is essential that academic health centres engage actively in ensuring that a culture of research inquiry is maintained*” ([37], p. 4)
Donovan et al., 2014 [38];Australia	National Breast Cancer Foundation – Wide range of programmes	Documentary analysis, bibliometrics, survey of PIs (242, 153 responded, 63%), 16 case studies, cross-case analysis Payback Framework	10% impact on policy – 29% expected to do so; 11% contributed to product development; 14% impact on practice/behaviour – 39% expected to do so	Basic research – more impact on knowledge and drug development; applied research – greater impact in other payback categories; many projects had only recently been completed – more impact expected; in launching the report the charity highlighted how it was informing their funding strategy [92]
Expert Panel for Health Directorate of the European Commission’s Research Innovation Directorate General, 2013 [39]; European Union	European Union Framework Programmes 5, 6, and 7 – Public health projects	Documentary review: all 70 completed projects; 120 ongoing; KI interviews with particularly successful and underperforming projects (16); data extraction form constructed based on the categories from the Payback Framework, with each of the main categories broken down into a series of specific questions Payback Framework	Appendix 1: only 6 out of the 70 completed projects did not achieve the primary intended output; 42% took actions to engage or inform policymakers; 4 (6%) projects change of policy, 22% expected to do so; 7 (10%) impact on health practitioners; 6 (9%) impact on health service delivery and 6 (9%) impact on health; 1 beneficial impact on small/medium-sized enterprise	Used documentary review, therefore for completed projects had data about whole set; however, “*Extensive follow-up of the post-project impact of completed projects was not possible*” ([39] p. 9) Comprehensive coverage of a programme without requiring additional data from the researchers; however, also shows the limitations of such an approach in capturing later impacts
Ferguson et al., 1998 [40]; United Kingdom	NHS Northern and Yorkshire Region – Health Services Research (HSR) (two other programmes not included here)	Desk analysis (bibliometrics), surveys to gather quantitative and qualitative data sent to all PIs and Co-Is in all three programmes: but only HSR projects asked about policy, so just the 32 HSR responses analysed here Refer to Payback Framework; no attempt to develop own	Five HSR projects (16%) had a policy impact, i.e. “*Better informed commissioning and contracting*” ([40] p. 17); 5 (16%) led to a change in NHS practice, i.e. “*More effective treatment, screening or management for patients*” ([40], p. 16)	This was part of a wider analysis, but in all three areas the projects were reactive; particularly difficult to make an impact with Primary and Community Care research
Gold & Taylor, 2007 [41]; United States of America	Agency for Healthcare Research and Quality – Integrated delivery systems research network	Documentary review of programme as a whole and individual projects (50); descriptive interviews (85); four case studies, additional interviews No explicit framework described	Changes in operations; “*Of the 50 completed projects studied, 30 had an operational effect or use*” [41] (Operational effect or use is a broad term: so the 60% put into our combined impact category)	Success factors: responsiveness of project work to delivery system needs, ongoing funding, development of tools that helped users see their operational relevance
Gutman et al., 2009 [42]; United States of America	Robert Wood Johnson Foundation – Active living research	A retrospective, in-depth, descriptive study utilising multiple methods; quantitative data derived primarily from a web-based survey of grantee investigators (PIs, Co-PIs), of the 74 projects: 68 responses analysed; qualitative data from 88 interviews with KIs The conceptual model used in the programme “*was used to guide the evaluation*” ([42], p. S23). Aspects of Weiss's model used for analysing policy contributions	Generally thought to be too early for much policy impact, but 25% of survey, 43% of interviewees reported a policy impact; however, policy impact in survey could be from active living research in general, not just the specific programme, and could include: “*a specific interaction with policymakers (e.g. testifying, meeting with policymakers, policymaker briefings, etc.) or direct evidence of the research findings in a written policy*” ([42], p. S33)	Only 16% of grants had been completed prior to the year of the evaluation; some approaches “*worked well, including developing a multifaceted, ongoing, interactive relationship with advocacy and policymaker organizations*” ([42], p. S32); grantees who completed both interviews and surveys generally gave similar responses, but researchers included in the random sample of interviewees gave higher percentage of policy impact than researchers surveyed; questions slightly different in the interviews than in the surveys
Hailey et al., 1990 [43]; Australia	National Health Technology Advisory Panel – HTA reports	Looked at technologies (20) covered by HTA reports from the panel up to end of 1988. Little provided on methods – presumably desk analysis, just states comparing recommendations, assessments and policy activities No framework described	Out of the first 20 technologies covered by HTA reports there had been significant impact in 11 and probable influence in three: 70% in total	Timing crucial factor for impact; at the margin of our inclusion criteria since it is based more on panel recommendations than a programme of research, but took first 20, not a selection
Hailey et al., 2000 [44]; Canada	Canadian province (not stated) – HTA brief tech notes	Interviews with those requesting the 20 brief HTA notes (i.e. reviews); checks on quality of the reports made using desk analysis and comments from experts No framework described	14 (70%) had influence on policy and other decisions	These HTA brief reviews were directly and urgently requested by users; at the margin of our inclusion criteria since it is not clear to what extent it was a research programme
Hanney et al., 2007 [6]; United Kingdom	National Health Service (NHS) – HTA programme	Multiple methods: literature review, funder documents, survey all PIs of projects between 1993 and 2003 (204, 133 responses, 65%), case studies with interviews (16) Payback Framework	Technology Assessment Reports (TARs) produced for the National Institute for Health and Clinical Excellence (NICE): 96% impact on policy, 60% on clinician behaviour; primary and secondary HTA research: 60% impact on policy, 31% on behaviour Average for programme: 73% impact on policy, 42% on behaviour; case studies showed large diversity in levels and forms of impacts and the way in which they arise	Different parts of the programme had different impact levels; key factors in achieving impact – agenda setting to meet needs of healthcare system, generally high scientific quality of research, existence of a range of ‘receptor bodies’ to receive and use findings, especially demonstrated for the NICE TARs; pre- and post-interview scoring showed reasonable correlations: suggests most survey responses not making exaggerated impact claims
Hanney et al., 2013 [45]; United Kingdom	Asthma UK – All programmes of Asthma research	Survey of all PIs (153, 96 responses, 59%), documents, case studies (14) involving interviews and some expanding the approach to cover role of chairs and centre Payback Framework	13% impact on policy; 17% product development; 6% health gain; but case studies reveal some important examples of influence on guidelines, some potentially major breakthroughs in asthma therapies, establishment of pioneering collaborative research centre	Many types of research and modes of funding – long-term funding of chairs led to important impacts; comparison of evidence from surveys and case studies on same projects showed generally exaggerated claims not made; study informed strategy of the medical research charity
Hera, 2014 [46]; Africa	Africa Health Systems Initiative Support to African Research Partnerships	Documentary review; interviews at programme level; project level information – for six projects, workshops, for the remaining four a total of 12 interviews; participant observation of end-of-programme workshop and presented some preliminary findings Key element of the design – adoption of an interactive model of knowledge translation	Policy impact was created during the research process: 7 out of 10 projects reported policy impact already, “*The policy dialogue is not yet complete and further uptake can be anticipated*” ([46], p. 3)	“*Research teams who started the policy dialogue early and maintained it throughout the study, and teams that engaged with decision-makers at local level, district and national levels simultaneously were more successful in translating research results into policy action*” ([46], p. 1); timing of evaluation – too early for some impact, but programme’s interactive approach led to some policy impact during project
Jacob & Battista, 1993 [47]; Quebec, Canada	Quebec Council on Health Care Technology Assessments (CETS) – HTA	Case study analyses of impact on decision-making and cost savings of reports in first 4 years (10) Scored for policy influence – critical incidents used Interviews (45) with scientific and political partners, and staff at CETS; documentary analysis also used Desk analysis of cost savings Developed own CETS approach	Examined impact on decision-making and cost savings; 8 of 10 reports influenced decisions	Identified a series of key features of the Quebec system that were favourable to HTAs making an impact; these include “*A general receptivity to rationality in decision making…the health care system in Quebec is organized in such a way that information produced by the council can filter easily into the decision-making process*” ([47], p. 571); this is an example of the receptor body playing an important role
Jacob & McGregor, 1997 [48]; Quebec, Canada	Quebec Council on Health Care Technology Assessments (CETS) – HTA	Comprehensive case study approach; similar to above on 21 reports in circulation sufficiently long for at least some impact to be estimated Used own CETS approach	18 of 21 reports influenced policy (86%); 8 at the highest level	Context was same as above; “*The best insurance for impact is a request by a decider that an evaluation be made*” ([48], p. 78) (not entirely clear if these 21 reports included 10 reports above)
Johnston et al., 2006 [49]; United States of America	National Institute of Neurological Disorders and Stroke – All pre 2000 phase III clinical trials in this field	Data on the effects of all 28 trials from desk analysis involving reviews, contact with PIs and others, and opinions of experts (4) Health economic modelling used to estimate return on investment (ROI) ROI analysis – a key example of a monetisation study	Six trials (21%) led to improvements in health: 470,000 quality-adjusted life years in 10 years since funding of 28 trials at cost of $3.6bn; the projected net benefit was $15.2bn; yearly ROI 46% (in total 8 studies, 29%, were identified as providing impact used in the analysis: two were cost savings only)	The main purpose of this study was to assess the public ROI; however, it seems to be the only such study that attempted to identify whether any health improvements had resulted from each individual project in a programme (and it thus met our inclusion criteria)
Kingwell et al., 2006 [50]; Australia	National Health and Medical Research Council (NHMRC) – Wide range of fields	Survey of all contactable PIs completing in 1997 using a simplified version of NHMRC end-of grant report as the survey instrument (259, 131 responses, 51%) No explicit framework	9% affect health policy; 24% affected clinical practice, 14% public health practice (in our analysis used the 24% as not clear how many might be duplicates); commercial potential: 41%	Highlighted some projects with clinically relevant outcomes for showcasing to the community
Kwan et al., 2007 [51]; Hong Kong	Health and Health Services Research Fund – Range of fields	Adapted Payback survey sent to PIs of completed projects (205, 178 responses, 87%); statistical analysis including multivariate analysis Payback Framework	Use in policymaking, 35%; changed behaviour, 49%; health service benefit, 42%	Multivariate analysis found that investigator participation in policy committees as a result of the research and liaison with potential users were significantly associated with health service benefit, policy and decision-making, and change in behaviour; however, set out various limitations in the methods used
McGregor et al., 2005 [52]; Canada	HTA Unit of McGill University Health Centre, Quebec – HTA	16 HTA reports examined; no account of concepts or techniques; presumably desk analysis of documents, etc. and insider account and informed by previous studies in Quebec (see Jacob [47] and [48] above)	All 16 (100%) HTA reports incorporated into hospital policy and some cost savings	Hospital’s HTA Unit combined researchers to synthesise evidence and a policy committee to make recommendations; success because “*(i) relevance (selection of topics by administration with on-site production of HTAs allowing them to incorporate local data and reflect local needs), (ii) timeliness, and (iii) formulation of policy reflecting community values by a local representative committee*” ([52], p. 263)
Milat et al., 2013 [53]; Australia	New South Wales Health Promotion Demonstration Research Grants Scheme	Semi-structured interviews with Chief Investigators (CI) (17) and end-users (29) of the 15 projects; thematic coding of interview data and triangulation with other data sources to produce case studies for each project Case studies individually assessed against four impact criteria and discussed to reach group assessment consensus Banzi Research Impact Model	10 out of 15 (67%) were in the moderate or high categories for impact on policy and practice combined (we did not have an economic category in our analysis and therefore decided not to include the combined health, social and economic category where 33% of the projects were rated as resulting in moderate or high impact)	High impact projects' success: “*due to the nature and quality of the intervention itself…, high quality research, champions who advocated for adoption, and active dissemination strategies. Our findings also highlight the need for strong partnerships between researchers and policy makers/practitioners to increase ownership over the findings and commitment to action*” ([53], p. 14)
Molas-Gallart et al., 2000 [54]; United Kingdom	Economic and Social Research Council AIDS Programme – Social aspects of AIDS	43 interviews with researchers of all 14 completed projects, then snowball approach for users: mapped network of researchers and users and post-research activity Framework based on the interconnection of three major elements: the type of output, the diffusion channels and the forms of impact – later contributed to development of Social Impact Assessment Methods through the study of Productive Interactions [23]	50% of researchers claimed programme provided non-academics with tools to solve problems and been used to develop policies	Concludes a two to three stage process required to assess impact (interview researchers first, then users); normal sampling techniques inadequate because impact not distributed along a normal distribution curve; detailed project-by-project qualitative analysis important
Oortwijn et al., 2008 [55]; The Netherlands	ZonMw Health Care Efficiency Research Programme – HTA	Survey data collected from PIs (43, 34 responses, 79%); case study analysis (including 14 interviews) of five HTA projects; developed and applied a 2-round scoring system Payback Framework	10 projects (29%) had a policy impact, including 6 being cited in guidelines; 11 projects (32%) reported implementation of new treatment strategies: counted as informed practice	The assessment was perhaps too soon after completion of the projects to witness benefits for many of projects; unlike most HTA programmes this had a large responsive mode element and most studies were prospective clinical trials
Poortvliet et al., 2010 [56]; Belgium	The Belgium Health Care Knowledge Centre (KCE) – HTA, HSR and good clinical practice	Documentary review; two group discussions: with 11 KCE experts, with 2 KCE mangers; interviews with stakeholders (20); web-based survey – total of 88 managers reported on 126 projects; nine detailed case studies; international comparisons with three agencies using documentary/literature review and interviews (3) Developed own framework	58% of project coordinators thought the project contributed to policy development: more for HTA than good clinical practice or HSR; 16 of the 20 stakeholders said findings influenced decision making, four said not in their organisation; 30% coordinators thought the project contributed to changes in healthcare practice	Factors linked to impact include involvement from “*stakeholders in agenda and priority setting. The quality of KCE research itself is high and in general beyond discussion. The relevance of KCE research findings is generally judged as high*” ([56], p. 111–2); some similarities with other/earlier findings about HTA being more likely to make impact
Reed et al., 2011 [57]; Australia	Primary care research	Online survey to 41 contactable CIs (out of 59 projects); asked impacts expected, how many achieved; some projects excluded as still underway, other refused; 17 completed out of 27 eligible Payback Framework	Four projects (24%) influenced national/state policymaking, but 8 (47%) influenced decision making at organisational, local or regional level (combined nine separate projects (53%) had policy/organisational decision impact); despite further examples of quite high levels of impact, surveys showed “*these perceived impacts affected the health service organizations, clinicians and patients who took part in the research projects*” ([57], p. 4) (we included the lowest of the three figures given for this, 29%)	The high level of use for policy and organisational decision making “*reflects a high level of engagement of the researchers with potential users of their research findings*” ([57], p. 5)
RSM McClure Watters et al., 2012 [58]; Northern Ireland, United Kingdom	Northern Ireland Executive: Health and Social Care Research – All fields	Desk analysis of documents and literature, consultations with stakeholders, survey informed by Payback Framework, three case studies, benchmarking. Surveys to all PIs for projects funded between 1998 and 2011 who could be contacted (169; 84 responses, 50%) Payback Framework	19% impact on policy development; for impact on health and the healthcare system: 20% health gain; 14% improvements in service delivery; 17% increased equity (the 20% figure used in our analysis represents the most conservative overall figure); substantial leveraged funds for follow-on projects came from outside Northern Ireland	Because Northern Ireland’s government did not contribute to the United Kingdom’s NIHR, researchers were not able to apply to the NIHR programmes. This “*was seen by respondents as a major constraint to research activity… research was not seen as a priority within many organisations and that many key stakeholders in the health sector did not fully engage with research or see its benefits*” ([58], p. 49); as a result of the assessment, Northern Ireland decided to subscribe to the NIHR
Sainty, 2013 [59]; United Kingdom	UK Occupational Therapy Research Foundation – Occupational therapy	PIs of completed project invited to complete a ‘personalized impact assessment form’ (equivalent to a survey) (11, 8 responded, 73%) Two researchers provided an independent review of the collated findings Becker Medical Library Model	Three projects (37.5%) reported local clinical application: “*particular tools, clinical advice, or models that were the subject of research having been used in practice*” ([59], p. 534)	In relation to the clinically related activities of three projects: “*Important to note, was the extent to which respondents highlighted this as being in the context of the participating services or host organizations*” ([59], p. 534)
Shah & Ward, 2001 [60]; Australia	NHMRC – Public health R&D committee	Self-complete questionnaires to CIs funded in 1993 (55, 38 responses, 69%); combined with desk analysis – attempted some correlations between publications and impact No framework stated	58% claimed research influenced policy; 69% influence on practice; 53% stated both	“*Influence on policy, practice or both was not associated with peer-reviewed publication in an Australian journal*” ([60], p. 558)
Soper & Hanney, 2007 [61]; United Kingdom	NHS Implementation Methods Programme – Implementation research	Postal survey of PIs (36, 30 responses, 83%) and potential users of the three projects in maternity care (227, 100 responses, 44%); poor response from other users to electronic survey; some desk analysis; interviews with key figures Payback Framework	30% claimed impact on policy; 27% on practice; 54% of the midwives and perinatal care researchers surveyed said the findings from at least one of the three maternity care projects had influenced their clinical practice	In this new field, the programme generated considerable enthusiasm among members of advisory and commissioning groups, and increased understanding and interest in the field; some projects made considerable impact, but IMP did not have a communications strategy and as a programme it highlighted some of complexities facing implementation.
The Madrillon Group, 2011 [62]; United States of America	NIH – Mind body interactions and health program	Mixed methods cross-sectional evaluation design; semi-structured interviews with 100% response rate – PIs of all 44 investigator-initiated projects and all 15 centres; impacts of centres scored by adapting the scales used previous in payback studies Adapted version of Payback Framework	Projects: 34% influenced policies; 48% led to improved health outcomes; the centres and projects, “*produced clear and positive effects across all five of the Payback Framework research benefits categories*” ([62], p. xiii)	Some projects were still in progress and it was too early to capture all the ‘latent’ impacts; conducted innovative analysis through examining three overlapping levels (programme, centre and projects); for assessing all projects used semi-structured interviews rather than surveys
Wisely, 2001 [63]; United Kingdom	NHS – National R&D programme on primary/secondary care interface	Survey of PIs of projects completed by April 2001 (63, 40 responded, 63%); desk analysis comparing grades for applications and quality of project Payback Framework	35% used in policy/decision making; 27% led to changes in practice; 25% health service benefits arisen	Some indication from limited data that applications graded as excellent more likely to lead to high quality projects with impact
Wisely, 2001 [64]; United Kingdom	NHS – National R&D programme, mother & child care	Survey of PIs of projects completed by April 2001 (39, 26 responded, 67%) Payback Framework	27% used in policy/decision making; 31% led to changes in practice; 23% health service benefits arisen	Some PIs thought that being part of a national R&D programme helped give their project greater credibility in the eyes of potential users
Wooding et al., 2009 [65]; United Kingdom	Arthritis Research Campaign – Wide range of arthritis research	Web-based tick list survey of PIs in 2007 of grants ending in 2002 and 2006 (136, 118 responses, 87%) Developed from the Payback Framework was subsequently named the RAND/ARC Impact Scoring System	6 projects (5%) policy impact; 8% “*generated intellectual property that has been protected or is in the process of being so*” ([65], p. 37) (over 80% of grants generated new research tools)	Much of the research funded was more basic and likely to inform further research rather than directly lead to impacts; also, it was probably too soon after the end of the projects to capture all the impact that might arise
Zechmeister & Schumacher, 2012 [66]; Austria	Institute for Technology Assessment and Ludwig Boltzmann Institute for HTA – HTA	Desk analysis identified all HTA reports aimed at use in re-imbursement or for disinvestment – 11 full HTA reports, 58 rapid assessments Descriptive quantitative analysis of administrative data and 15 interviews with administrators and payers Analysis informed by Quebec studies – see above, Jacob [47] and [48]	Five full HTA reports and 56 rapid assessments “*were used for reimbursement decisions*”, four full HTAs and two rapid assessments “*used for disinvestment decisions and resulted in reduced volumes and expenditure*” ([66], p. 77) Total of 67 out of 69 used (97%); two full HTAs no impact; other factors also played a role: in only 45% of reports “*the recommendation and decisions totally consistent*” ([66], p. 81)	In Austria, policymaking structures facilitate the use of HTA reports, but no mandatory requirement to do so; it is possible the decisions could have been made based on international HTA institutions, but unlikely because, to be used, HTA reports “*need primarily to be in German language and they have to be produced within a time period that is strongly linked to the decision-making process*” ([66], p. 77)

The studies came from 11 different countries, plus a European Union study and one covering various locations in Africa. The number of projects supplying data to the studies ranged from just eight in a study of an occupational therapy research programme in the United Kingdom [59], to 22 operational research projects in Guatemala [35], 153 projects in a range of programmes within the portfolio of the Australian National Breast Cancer Foundation [38], and 178 projects from the Hong Kong Health and Health Services Research Fund [51].

In terms of the methods used to gather data about the projects in a programme, 21 of the 36 studies surveyed the researchers, usually just each project’s Principal or Chief Investigator (PIs), either as the sole source of data or combined with other methods such as documentary review, interviews and case studies. Six studies relied exclusively, or primarily, on documentary review and desk analysis. In at least three studies, interviewing all PIs was the main method or key starting point used to identify further interviewees. The picture is complicated because some studies used one approach, usually surveys, to gain information about all projects, and then supplemented that with other approaches for selected projects on which case studies were additionally conducted, and often involved interviews with PIs. In total, over a third of the studies involved interviews with stakeholders, again sometimes in combination with documentary review. Many studies drew on a range of methods, but two examples illustrate a particularly wide range of methods. In the case of Brambila et al. [35] in Guatemala, this included site visits which were used to support key informant interviews. Hera’s [46] assessment of the impact of the Africa Health Systems Initiative Support to African Research Partnerships involved a range of methods. These included documentary review, and programme level interviews. Project level information was obtained from workshops for six projects and from a total of 12 interviews for the remaining four projects. In addition, they used participant observation of an end-of-programme workshop, at which they also presented some preliminary findings. In this instance, while the early timing of the assessment meant that it was unable to capture all the impact, the programme’s interactive approach led to some policy impact during the time the projects were underway.

In 20 of the 36 studies, the various methods used were organised according to a named conceptual framework (see Hanney et al. [6] and Raftery et al. [9] for a summary of all these frameworks); 16 of the 36 studies drew partly or wholly on the Payback Framework [15]. A series of existing named frameworks each informed one of the 36 studies, and included the Research Impact Framework [24], applied by Caddell et al. [37]; the Canadian Academy of Health Sciences framework [10], applied by Adam et al. [32]; the Banzi Research Impact model [11], applied by Milat et al. [53]; and the Becker Medical Library model [67], applied by Sainty [59].

In addition, various studies were identified as drawing, at least to some degree, on particular approaches, albeit without an explicitly named framework being described. Jacob and Battista [47] developed and applied their own approach to evaluate the impact of studies conducted by the Quebec Council of Health Care Technology Assessments (CETS); the approach was broadly replicated in a further evaluation of the impact from CETS [48] and informed subsequent studies in Quebec [52], France [34] and Austria [66]. The interactive approach was referred to by several studies [35, 46]. The study by Molas-Gallert et al. [54] of the impact from a programme of AIDS research funded by the United Kingdom’s Economic and Social Research Council used an approach that they subsequently further developed with Spaapen et al. [23] in the Social Impact Assessment Methods through the study of Productive Interactions (SIAMPI) approach.

Only one included study assessed the monetary value of a research programme’s resultant health gain. Johnston et al.’s [49] assessment of the impact from a National Institutes of Health (NIH) programme of clinical trials in the United States is described in some detail here because studies providing a rate of return were seen in the World Health Report as key evidence for promoting the future funding of health research [1]. For the trials identified as making an impact in terms of health gain and/or cost savings, Johnston et al. [46] employed a bottom-up approach. They identified cost-utility estimates for the interventions implemented following the NIH research to obtain a per patient net monetary benefit. A timeline of usage was constructed for each of the interventions to produce a population timeline of net monetary benefit and was related to the investment in research. The results indicated an impact, with a return on investment for the whole programme of 46% per year. However, the authors acknowledged the difficulty of acquiring the necessary data to conduct an exercise of this kind, with only 8 out of 28 trials contributing the benefits used to calculate the rate of return on investment. While we did not have a category related specifically to the economic impacts of health research, we included this study in the health gain category because the latter was a key step towards being able to calculate monetary value and was identified as occurring in six out of the 28 projects (21%).

Despite the diversity, each of the 36 studies reported on the number of projects in the multi-project programme making an impact in one, or more, of four broad categories. The number of projects reporting on each category, and the number (and range) of projects that reported having achieved some such impact is set out in Table 2.

**Table 2:** Analysis of quantitative data from 36 studies reporting on findings from each project in a multi-project programme

Type of impact	Out of 36 studies number reporting on each impact category	Median (range) percentage achieving/claiming this impact in the studies reporting on it
Policy/organisation impact	31	35% (5–100%)
Clinician change/informed practice	17	32% (10–69%)
A combined category, e.g. policy and clinician impact, or impact on decision-making	3	64% (60–67%)
Health gain/patient benefit/improved care	12	27% (6–48%)

One example from the various studies can be used to illustrate what is included in each of the four types of impact. The 1997 study by Jacob and McGregor [48] reported that 86% of the HTAs conducted in Canada by the Quebec CETS had influenced policy. One of these HTAs found that the likelihood of health benefits from routine preoperative chest radiography was extremely slender; prior to the publication of that HTA report, 55 out of 118 hospitals questioned had a policy of using such routine chest radiography, yet 3 years later, all but three had abandoned this policy and in 79% of cases the HTA was cited as a reason for the policy change. In terms of impact on practice, in 2007, Kwan et al. gave the following as an example of the local impact on provider behaviour made by the health and health services research programme in Hong Kong: “*improved reporting of unintentional child injury cases and liaison between the Hospital Authority Informatics and Accident and Emergency*” ([51], p. 8).

Illustrating the combined category, Milat et al. [53] used a category called ‘Policy and practice impacts’ in their 2013 assessment of the impact from the research funded in Australia by the New South Wales Health Promotion Demonstration Research Grants Scheme. While the analysis provided overall figures only for this combined category, the few examples that were given were presented separately for policy impacts and practice impacts. In some, but not all, instances the accounts covered both dimensions, for example, research informed policy planning by identifying areas for investment in tai chi for older people (as a way of preventing falls) and smoking cessation brief interventions. Then, in terms of practice, the research in those same two areas helped inform professional development for the relevant staff providing the services. An example of health gain comes from one of the NIH trials analysed in the 2006 assessment by Johnston et al. [49] described above, where the authors estimated that implementation of the findings from the trial of the use of tissue plasminogen activator in cases of acute ischemic stroke, published in 1995, had a projected health gain in the 10 years after funding was completed of 134,066 quality-adjusted life years.

For each category, apart from the combined one, there was a wide range in the proportion of studies per programme that had demonstrated (or claimed) impact in each category.

Most included studies had considered key factors that might help explain the level of impact achieved (see last column in Table 1 for direct quotes, or comments that in most cases came from the original paper). Differences in impact appeared to relate partly to the approaches used and the timing of the assessment. For example, one study that appeared to shown a very low proportion of projects with impact on policy had assessed this purely through desk analysis of end-of-project reports. Such an approach restricted the opportunities to identify the actual levels of impact achieved, as opposed to the expected levels of impact, which were much higher and at least some of which would presumably have arisen later [39].

Various features of the different programmes of research also influenced the levels of impact achieved. In four studies of research programmes, 10% or fewer of PIs reported that their research had made an impact on policy, but three of these studies [38, 50, 65] included basic research (from which direct policy impact would be much less likely to occur) and, in two of those, assessment of impact was performed relatively soon after completion of the research.

While the median for the 31 studies reporting on policy impact made by programmes was 35% of projects making such an impact, the interquartile range was 20–70%. This reflects the existence of both a group of studies, as described above, where a very low proportion of projects informed policies, and a group of studies with a very high proportion of projects informing policies. In fact, a median of 77% (range 29–100%) of projects in the nine included HTA programmes [6, 34, 43, 44, 47, 48, 52, 55, 66] had had a demonstrable impact on policy. Even within this group of programmes, the type of research conducted varied. Most were technology appraisal reviews that had usually been requested by those making decisions for the relevant health service about funding (or disinvesting in) particular technologies or services. In some cases, an extremely high proportion of projects in these programmes made an impact on policy; for example, 97% of the assessments from the Austrian HTA programme were classified as making at least some impact on coverage policies [66], as were 100% of the HTA reports from the HTA unit of McGill University Health Centre in Quebec, Canada [52]. By contrast, while the Health Care Efficiency Research programme from the Netherlands was classified as an HTA programme, it included a large responsive mode element and most studies were prospective clinical trials and impact assessment occurred soon after the end of the trials [55]; a lower proportion of projects in these studies (29%) had demonstrated a policy impact.

The review of programmes funded in the first decade of the United Kingdom HTA Programme showed that, overall, 73% of projects had an impact on policy [6]. Of these, 96% of technology appraisal reviews undertaken to inform the work of the, then, National Institute for Health and Clinical Excellence, actually did so (that is, they were commissioned to inform the work of a specific user body), and 60% of other projects (mostly trials) had a direct impact on policy. The 60% figure for these latter studies compares favourably with the median of 35% in our sample overall, and is probably due to the fact that, even though the projects were not usually commissioned by a specific user body, they were on topics that had been identified as meeting a need within the healthcare system. In only four of the 22 non-HTA programmes that reported making an impact on policy was the claimed figure higher than 50% of projects [46, 56, 57, 60]. In three of those [46, 56, 57], the authors identified involvement of potential users in agenda setting and/or interaction over the research as a key factor facilitating impact. For example, Reed et al. said that the figure of 53% of projects from a programme of primary care research in Australia making an impact on policy and organisational decisions reflected “*a high level of engagement of the researchers with potential users of their research findings*” ([57], p. 5) (See Table 1 for further details).

Similarly, of the seven non-HTA programmes with a high proportion of projects making an impact in terms of informing practice or clinician behaviour, three highlighted the importance of interaction with potential users [32, 33, 51] and a further two were small-scale funding initiatives where the impact was often on clinicians at the location where the research had been conducted [37, 59]. In all three of the programmes where the impact was in the combined policy and practice category the proportion of projects making an impact was at least 60%, and there was interaction with users and/or the research met their needs [35, 41, 53].

Finally, in some instances observations were recorded on how the impact evaluations of whole programmes of work had been, or could be, used to inform policies of the research funding body whose work had been assessed and/or used to highlight the benefits that arise from donating to medical research charities. Examples include public research funders, such as the Catalan Agency for Health Information, Assessment and Quality, and the Northern Ireland Executive [32, 58], and medical research charities such as Asthma UK and the Australian National Breast Cancer Foundation [38, 45].

## Discussion

The findings provide lessons about how a range of methods for assessing research impact can be applied, with surveys of PIs being the most frequently used, but interviews and desk analysis also being adopted as alternatives or supplements. Such methods could be adopted elsewhere in future research impact assessments. Furthermore, the methods adopted and the whole impact study were often, but not always, organised using an existing conceptual framework. The various approaches used in impact assessments have different strengths and weaknesses, and a range of theoretical underpinnings. A selection of six key established frameworks was analysed in Greenhalgh et al. [8], namely the Payback Framework [14], the Research Impact Framework [24], the Canadian Academy of Health Sciences framework [10], monetary value approaches [68], social impact assessment [23, 69] and the Research Excellence Framework (REF) [70], a pioneering approach used in the United Kingdom to assess the impact from university research groups and on which considerable subsequent analysis has been conducted [71]. While the approach used in the REF is not related to specific programmes of research, but to the research of teams who often had multiple sources of funding, the REF built on approaches originally developed to assess the impact of research programmes. The first five of the six frameworks highlighted by Greenhalgh et al. [8] helped inform at least one of the 36 studies in this current analysis and, according to the Higher Education Funding Council for England, the sixth (i.e. the REF) was itself partly informed by studies applying the Payback Framework [72]. These six key frameworks are described in Box 2.

**Box 2** Summary of major impact assessment frameworks

**Table:** 

**The Payback Framework**
Developed by Buxton and Hanney in 1996, the Payback Framework consists of two elements, namely a logic model of the seven stages of research from conceptualisation to impact and five categories to classify the paybacks [14]: • knowledge (e.g. academic publications) • benefits to future research (e.g. training new researchers) • benefits to policy (e.g. information base for clinical policies) • benefits to health and the health system (including cost savings and greater equity) • broader economic benefits (e.g. commercial spin-outs) Two interfaces for interaction between researchers and potential users of research (‘project specification, selection and commissioning’ and ‘dissemination’) and various feedback loops connecting the stages are seen as crucial. The Payback Framework can be applied through surveys, which can be applied to all PIs but have various limitations or to case studies. For the latter, researcher interviews are combined with document analysis and verification of claimed impacts to prepare a detailed case study containing both qualitative and quantitative information; this provides a fuller picture than surveys, but is more labour intensive. **Research Impact Framework (RIF)** Originally developed by Kuruvilla et al. [24] for academics who were interested in measuring and monitoring the impact of their own research, RIF is a ‘light touch’ checklist intended for use by individual researchers who seek to identify and select impacts from their work. Categories include • research-related impacts • policy and practice impacts • service (including health) impacts • ‘societal impact’ (with seven sub-categories) Because of its (intentional) trade-off between comprehensiveness and practicality, it generally produces a less thorough assessment than the Payback Framework and was not designed to be used in formal impact assessment studies by third parties. However, the approach proved to be highly acceptable to those researchers with whom it was applied. **Canadian Academy of Health Sciences (CAHS) Framework** CAHS Framework was developed from the Payback Framework through a multi-stakeholder consensus-building process; it is claimed to be a ‘systems approach’ that takes greater account of non-linear influences [10]. It encourages a careful assessment of context and the subsequent consideration of impacts under five categories: • advancing knowledge (measures of research quality, activity, outreach and structure) • capacity building (developing researchers and research infrastructure) • informing decision-making (decisions about health and healthcare, including public health and social care, decisions about future research investment, and decisions by public and citizens) • health impacts (including health status, determinants of health – including individual risk factors and environmental and social determinants – and health system changes) • economic and social benefits (including commercialisation, cultural outcomes, socioeconomic implications and public understanding of science) For each category, a menu of metrics and measures (66 in total) is offered, and users are encouraged to draw on these flexibly to suit their circumstances. By choosing appropriate sets of indicators, CAHS can be used to track impacts within any of the four ‘pillars’ of health research (basic biomedical, applied clinical, health services and systems, and population health – or within domains that cut across these pillars) and at various levels (individual, institutional, regional, national or international). **Monetisation models** Monetisation models, which are mostly at a relatively early stage of development [68], express returns on research investment in various ways, including as cost savings, the monetary value of net health gains via cost per quality-adjusted life year using metrics such as willingness-to-pay or opportunity cost, and internal rates of return (return on investment as an annual percentage yield). These models draw largely from the economic evaluation literature and differ principally in terms of which costs and benefits (health and non-health) they include and in the valuation of seemingly non-monetary components of the estimation. Prevailing debates on monetisation models of research impact centre on the nature of simplifying assumptions in different models and on the balance between ‘top down’ approaches (which start at a macro level and consider an aggregate health gain, usually at a national level over a specific period, and then consider how far a (national) body of research might have been responsible for it arising) or ‘bottom-up’ approaches (which start with particular research advances, sometimes all the projects in a specific programme, and calculate the health gain from them). **Societal impact assessment (SIA)** Used mainly in the social sciences, SIA emphasises impacts beyond health. Its protagonists distinguish the social relevance of knowledge from its monetised impacts, arguing that the intrinsic value of knowledge may be less significant than the varied and changing social configurations that enable its production, transformation and use. Assessment of SIA usually begins by self-evaluation by a research team of the relationships, interactions and interdependencies that link it to other elements of the research ecosystem (e.g. nature and strength of links with clinicians, policymakers and industry), as well as external peer review of these links. SIA informed the Evaluating Research in Context programme that produced the Sci-Quest model [69] and also the EU-funded SIAMPI (Social Impact Assessment Methods through the study of Productive Interactions) framework [23]. Sci-Quest was described by its authors as a ‘fourth-generation’ approach to impact assessment – the previous three generations having been characterised, respectively, by measurement (e.g. an unenhanced logic model), description (e.g. the narrative accompanying a logic model) and judgement (e.g. an assessment of whether the impact was socially useful or not). Fourth-generation impact assessment, they suggest, is fundamentally a social, political and value-oriented activity and involves reflexivity on the part of researchers to identify and evaluate their own research goals and key relationships [69]. Whilst the approach has many theoretical strengths, it has been criticised for being labour intensive to apply and difficult to systematically compare across projects and programmes. **United Kingdom Research Excellence Framework (REF)** The 2014 REF – an extensive exercise developed by the Higher Education Funding Council for England to assess United Kingdom universities’ research performance – allocated 20% of the total score to research impact [70]. Each institution submitted an impact template describing its strategy and infrastructure for achieving impact, along with several four-page impact case studies, each of which described a programme of research, claimed impacts and supporting evidence. These narratives, which were required to follow a linear and time-bound structure (describing research undertaken between 1993 and 2013, followed by a description of impact occurring between 2008 and 2013) were peer-reviewed by an intersectoral assessment panel representing academia and research users (industry and policymakers). Almost 7000 impact case studies were produced for the 2014 REF; these have been collated in a searchable online database on which further research is currently being undertaken [71]. Independent evaluation by RAND concluded that the narrative form of the REF impact case studies and their peer review by a mixed panel of experts from within and beyond academia had been a robust and fair way of assessing research impact. In its internal review of the REF, the members of Main Panel A, which covered biomedical and health research, noted that “*International MPA* [Main Panel A] *members cautioned against attempts to ‘metricise’ the evaluation of the many superb and well-told narrations describing the evolution of basic discovery to health, economic and societal impact*” [70].

One of the featured approaches currently receiving more attention is the attempt to put a monetary value on the impact of health research, and in particular studies involving attempts to value the health gain from research. Various examples of the latter were identified in the two reviews [73–79]. One study, that of Johnston et al. [49], occupies a particular place in the consideration of frameworks because it included all individual projects within a programme (see above) and, while all of the projects were examined, only a small proportion were identified as making a measurable impact. Those projects ensured the programme as a whole had a high rate of return. Some other studies with a more limited scope have also used a bottom-up approach to assess the impact of specific projects, but have not gone as far as attempting a comprehensive valuation of the impact of a whole programme of research. Nevertheless, such studies can indicate probable minimum levels of returns from the whole programme studied [79].

It is important to acknowledge that this review has a number of limitations. First, fine distinctions had to be made about which studies to include, and some studies that initially seemed relevant had to be excluded because data extracted could not be meaningfully combined with those of other studies, thus reducing the comprehensiveness of the review. The seven studies [80–86] assessing the impact of multi-project programmes that were included in the two reviews on which this study was based, but excluded from this current analysis, are listed on Table 3, along with reasons for their exclusion.

**Table 3:** Seven excluded studies

Author, date, location	Programme/speciality	Reason for exclusion
Alberta Heritage Fund for Medical Research, 2003 [80]; Alberta, Canada	Alberta Heritage Fund for Medical Research HTA programme	The number of projects in which any impact (only on policy) was identified was described as ‘most’, which could not be included in the statistical analysis (NB: this is a different study than the one with the same author and same year that was included in the analysis as reference [33])
Aymerich et al., 2012 [81], Spain	Network centre for research in epidemiology and public health	Data for impact on reviews and on guidelines/other policies was combined making it impossible to identify the specific policy impact that would have been made by the contribution to guidelines, etc.; the healthcare benefits were potential not actual
Catalan Agency for HTA and Research, 2006 [82], Catalonia, Spain	TV3 telethon for biomedical research in Catalonia: different speciality each year	Most of the data on impacts seemed to be potential impacts, and the data that were available were presented as total instances not the percentage of projects reporting the impact category
Cohen et al., 2015 [83], Australia	National Health and Medical Research Council: intervention studies in various programmes	While it was a multi-project assessment covering 70 eligible intervention projects, they came from more than one programme and were not the total number of projects from the programmes of which they were part
NHS Executive Trent, 1997 [84], United Kingdom	Programme of the Trent Region of the NHS: wide range of basic and applied research	The number of projects in which any impact (on policy and on practice) was identified was described just as ‘<10’, and so not included in the statistical analysis
Shani et al., 2000 [85], Israel	Israeli Ministry of Health’s Medical Technologies Administration/Israeli Center for Technology in Health Care: HTA	The number of projects in which any impact (only on policy) was identified was described just as ‘86–100’, and so not included in the statistical analysis; also the paper was a commentary rather than a research report
Stryer et al., 2000 [86], United States of America	Agency for Health Care and Research Quality: Outcomes and effectiveness research	The number of projects in which any impact (on policy and on practice) was identified was described as ‘limited’, and so could not be included in the statistical analysis

Second, each of the included studies was liable to have inherent weaknesses associated with the type of data gathering techniques employed in assessing impact from multi-project programmes. Many of the studies relied on self-reported survey data, and some of them acknowledged potential concerns about such data [51]. Nevertheless, approaches such as triangulation can somewhat mitigate these weaknesses and, in at least four examples, data were collected both by surveys and interviews and, in each case, the self-reported survey data did not seem, on average, to over-emphasise the level of impact [6, 36, 42, 45]. A further limitation with surveys is that the response rate was generally between 50% and 75%, with only four studies receiving replies from more than three-quarters of projects: Kwan et al. [51], 87%; Oorwijn et al. [55], 79%; Soper and Hanney [61], 83%; and Wooding et al. [65], 87%. Other approaches, such as the desk analysis based on end of project reports [39], obtained data from a higher proportion of projects, but, as described above, provided limited opportunities to gather data on actual impacts achieved. To the extent that differences in the impact identified for each programme reflect differences in the approach used to conduct the assessment, there will be limitations in drawing lessons from the overall dataset of 36 assessments of the impact from programmes.

Third, in various studies, it was observed that the impact assessment was occurring at a time that was too early for some, or most, of the research to have had time to make an impact [38, 39, 42, 55, 65]. In such cases, the reported level of impact achieved was not only likely to be lower than it would have been in a later assessment, but also it might look comparatively lower than that from other programmes included in the analysis where the assessment took place some years after the research had been completed. This again complicates attempts to draw lessons from the overall dataset of 36 programmes.

Fourth, in order to facilitate the analysis, it was necessary to create a small number of impact categories, but the definitions for impact categories used in the diverse studies varied widely. Therefore, compromises had to be made and not all the examples included in each category had been defined in precisely the same way; therefore, what was included in a category from one study might not exactly match what was included in the same category from another study. Particular problems arose in relation to whether there should be a ‘cost-savings’ category. There has been considerable debate about the place for cost-savings within an impact categorisation [9]; it was decided not to include a separate cost-saving category in this current analysis. However, various studies had cost-savings as one element in the broader category of ‘impact on health gain, patient benefit, improved care or other benefits to the healthcare system’ and these were included.

A final limitation is that each project counted equally to the final tally, and the question of whether impact had occurred was framed as a binary yes/no. This meant that large, well-conducted projects that had produced very significant impacts counted the same as smaller, more methodologically questionable projects whose impact was limited (but which could still be said to have occurred). In quite a few of the individual impact assessments this limitation was reduced because more detailed case studies were also conducted on selected case studies. These were often reported to provide examples of the significant impact. However, in our current analysis, any supplementary case studies were not included in the data used to construct Table 2, which is the main comparative account of the findings.

Given the various limitations, the findings should be viewed with a degree of caution. Nevertheless, the included studies do present evidence of wide-ranging levels of impact resulting from diverse programmes of health research. Quite large numbers of projects made at least some impact, and case studies often illustrated extensive impact arising from certain projects. Our findings resonate with theoretical models of research impact, namely impact is more likely to be achieved when the topics of applied research, and how it might best be conducted, are discussed with potential users of the findings and when mechanisms are in place to receive and use the findings [6, 13, 16–21, 28–30]. We also found variations depending on the nature of the research being conducted. These points can be illustrated by some of the more notable examples from Table 1. For example, in the case of 100% of HTA reports from the HTA unit of McGill University Health Centre in Quebec, Canada, the impact was said to be because of *“(i) relevance (selection of topics by administration with on-site production of HTAs allowing them to incorporate local data and reflect local needs), (ii) timeliness, and (iii) formulation of policy reflecting community values by a local representative committee*” ([52], p. 263). In the case of 97% of the assessments from the Austrian HTA programme being classified as making at least some impact on coverage policies [66], there were features of the Austrian policymaking structures that facilitated the use of HTA reports. The authors explained that, to be used, the HTA reports “*need primarily to be in German language and they have to be produced within a time period that is strongly linked to the decision-making process*” ([66], p. 77). By contrast, and as noted above, while the Health Care Efficiency Research programme from the Netherlands was also classified as an HTA programme, it included a large responsive mode element and most studies were prospective clinical trials rather than the technology appraisal reports that are the main element of many HTA programmes [55]. The lower proportion of projects in these studies (29%) demonstrating a policy impact illustrates that variations in levels of impact achieved can be linked to the type of research conducted, even in the same overall field, which in this case was further exacerbated by the impact assessment occurring soon after the end of the trials [55].

Overall, as Jacob and McGregor reported for the HTAs conducted in Canada by the Quebec CETS, “*The best insurance for impact is a request by a decider that an evaluation be made*” ([48], p. 78). Furthermore, for those programmes (or parts of wider programmes) for which there were explicit mechanisms such as formal committees to receive and use the findings from technology appraisal reports in coverage decisions about investment or disinvestment, the proportion of projects making an impact was very high.

Further examples of studies of the impact of multi-project programmes have been published since the second review was conducted, with the examples from Bangladesh, Brazil, Ghana and Iran [2–5] illustrating a widening interest in producing evidence of impact. In the Ghanaian example, 20 out of 30 studies were used to contribute to action, and Kok et al. again showed that considerable levels of impact could be achieved by adopting an interactive approach; they reported that “*the results of 17 out of 18 user-initiated studies were translated into action*” ([4], p. 1). These four impact assessments provide further evidence that contributes to the global pool of studies showing the breadth of impact made by health research, and also reinforces the evidence that research impact assessment has become a rapidly growing field.

As was noted, some individual studies provided lessons for the specific funder on whose research they focussed as to how that funder might best use its research resources. Some more general lessons could also be drawn in terms of the types of research programmes, for example, needs led and collaborative ones, that seem to be more likely to lead to impacts, though it is widely understood that overall it is desirable for there to be a diversity of health research funded. Additionally, the growing body of evidence about the impacts that come from health research could potentially be used to promote research funding along the lines argued in the World Health Report 2013 [1]. Studies showing the monetary value in terms of a high rate of return on health research expenditure, whether from specific programmes or more widely, seem to have particular potential to be used to promote the case for further funding for medical research [77].

Lessons can also be learnt from the review about the range of methods and frameworks available to conduct health research impact assessments. Furthermore, in addition to continuing refinement of existing frameworks, for example, of the Canadian Academy of Health Sciences’ framework in Canada [87], there are also ever-increasing numbers of studies on which to draw to inform analysis, including current work in Australia [88]. Given the expanding focus on research impact assessment, the potential lessons that could be drawn from them, individually and collectively, are likely to be more significant if there could be somewhat greater standardisation. Any standardisation of methods might attempt to reduce the current diversity on items such as the categories of impact to include and their definition, and the timing of data collection and its presentation. Such moves towards standardisation might facilitate comparisons between processes used in different programmes and, in that way, inform strategic decisions that funding organisations will always need to make as to how best to use resources.

Some ideas about standardisation, as well as some potential dangers, might come from recent experience in the United Kingdom where many research funders are now using a standardised approach called Researchfish® (Researchfish Ltd, Cambridge, United Kingdom). This is an on-line survey, originally developed with the United Kingdom’s Medical Research Council, that an increasing number of research funders are now sending annually to the PIs of all the projects they support. It asks for information on outputs, outcomes and impacts (see Raftery et al. [9] for a more detailed account). It has several advantages, including a high formal response rate, wide use that could facilitate comparability between programmes and funders (though it does not currently report data in a way that would have facilitated its use in the comparisons made in our analysis), and a database that builds up a fuller picture over successive years, including a number of years after a project’s competition, thus allowing the capture of certain data that a one-off bespoke survey might miss. Its main limitations include being a burden on researchers (although this has been reducing as successive versions of the assessment survey have been made more user-friendly), the potential danger of a poorer response rate to key questions than can be obtained by bespoke surveys, and reduced specificity for some aspects of health research because it has been standardised to cover many research fields. As with other survey approaches, Researchfish provides less detailed information and understanding than can come from case studies, but allows wider coverage for the same resources.

How best to address these issues when seeking more standardised approaches could be of interest to the newly established WHO Global Observatory for Health Research and Development [89]. Furthermore, perhaps there would be scope for bringing together the expanding body of evidence providing examples of the impact from programmes of health research, with the increasing sophistication, and global spread, of the analysis of factors that might be associated with research use [90, 91].

## Conclusion

The quite high proportion of projects that reported making an impact from some multi-project programmes, including needs-led and collaborative ones, as well as the demonstration of the monetary value of a programme, could potentially be used to promote future research funding along the lines argued in the World Health Report 2013 [1]. This review also indicates that the evidence about health research impact is continuing to grow.

In addition to being of value to research managers in identifying factors that might lead to increased impact, this review of impact studies also demonstrates the range of methods and conceptual frameworks that can be used in conducting such studies. However, weaknesses in some studies, and diversity between studies in terms of methods and timing used, reduces the value of some individual studies and the ability to make comparisons within the full suite of 36 studies.

A standardised approach to assessing the impact of research programmes could address existing methodological inconsistencies and better inform strategic decisions about research investment in order to enhance impact. However, experience from the United Kingdom shows that moving towards such standardisation can itself generate further difficulties. There could be a role for the newly established WHO Global Observatory for Health Research and Development [89] in both drawing on the existing evidence from many countries about the impact of health research and in promoting ideas for achieving greater standardisation in health research impact assessment.

## Additional file

Additional file 1: Literature search strategies for the two reviews. (PDF 242 kb)

## Abbreviations

CETS: Council of Health Care Technology Assessments (Quebec)
HTA: health technology assessment
NIH: National Institutes of Health
NIHR: National Institute for Health Research
REF: Research Excellence Framework.
