# Supplementary material for: The impact on healthcare, policy and practice from 36 multi-project research programmes: findings from two reviews
Source: Health Res Policy Syst. 2017 Mar 28;15:26. doi: 10.1186/s12961-017-0191-y (PMC5371238; doi:10.1186/s12961-017-0191-y)
Supplement: Additional file 1: — Literature search strategies for the two reviews. (PDF 242 kb) [file 12961_2017_191_MOESM1_ESM.pdf]

Additional file 1: Literature search strategies for the two reviews

Example of the search strategy used in the first review (MEDLINE):

1. health services research
2. ((health technolog$ adj3 assessment$) or hta).mp.
3. (NHS adj6 research$).mp. [mp=title, original title, abstract, name of substance word, subject heading word]
4. exp *Technology Assessment, Biomedical/
5. "research and development".mp.
6. Health Policy/
7. (payback or pay back).ti,ab.
8. (("benefit$" or "utili#ation" or "impact" or "influenc$" or "gain$2") adj4 (research or evidence or health technolog$)).ti,ab.
9. ((implement$ or disseminat$) adj4 (benefit$ or impact or gain$)).ti,ab.

Search terms (1 or 2 or 3 or 4 or 5 or 6) AND (7 or 8 or 9)

Limited to English language and years 1990–2005

Search strategy for the second review: Search date: August 2014

Database	Search Strategy	Download file
Ovid MEDLINE(R) without Revisions <1996 to August Week 2 2014><br><br>Saved as med-impact-final-strategy	1 Health Services Research/ (21575)<br>2 ((health technolog$ adj3 assessment$) or hta).mp. (2254)<br>3 (NHS adj6 research$).mp. [mp=title, abstract, original title, name of substance word, subject heading word, keyword heading word, protocol supplementary concept word, rare disease supplementary concept word, unique identifier] (316)<br>4 exp *Technology Assessment, Biomedical/ (2993)<br>5 *Biomedical Technology/ec, mt, og, sn, td [Economics, Methods, Organization & Administration, Statistics & Numerical Data, Trends] (848)<br>6 Health Policy/ (35849)<br>7 1 or 2 or 3 or 4 or 5 or 6 (60818)<br>8 (payback or pay back).ti,ab. (239)<br>9 (("benefit$" or "utili#ation" or "impact" or "gains" or "returns") adj5 (research or evidence or health technolog#)).ti,ab. (18997)<br>10 *"diffusion of innovation"/ (6104)<br>11 *Information Dissemination/mt [Methods] (1903)<br>12 *Program Evaluation/ (5976)<br>13 *Policy making/ (3089)<br>14 *Cost-Benefit Analysis/mt, sn, td [Methods, Statistics & Numerical Data, Trends] (607)<br>15 8 or 9 or 10 or 11 or 12 or 13 or 14 (36356)<br>16 15 and 7 (2923)<br>17 limit 16 to (english language and yr="2005 -Current")	494

	(1842)<br>18 limit 17 to (evaluation studies or "review" or systematic reviews) (494)	
Database: Ovid MEDLINE(R) In-Process & Other Non-Indexed Citations <August 13, 2014>	1 Health Services Research/ (0)<br>2 ((health technolog$ adj3 assessment$) or hta).mp. (524)<br>3 (NHS adj6 research$).mp. [mp=title, abstract, original title, name of substance word, subject heading word, keyword heading word, protocol supplementary concept word, rare disease supplementary concept word, unique identifier] (43)<br>4 exp *Technology Assessment, Biomedical/ (0)<br>5 *Biomedical Technology/ec, mt, og, sn, td [Economics, Methods, Organization & Administration, Statistics & Numerical Data, Trends] (0)<br>6 "research and development".mp. (975)<br>7 Health Policy/ (0)<br>8 1 or 2 or 3 or 4 or 5 or 6 or 7 (1531)<br>9 (payback or pay back).ti,ab. (31)<br>10 (("benefit$" or "utili#ation" or "impact" or "gains") adj5 (research or evidence or health technolog#)).ti,ab. (2857)<br>11 ((implement$ or disseminat$) adj2 (benefit$ or impact or gain)).ti,ab. (167)<br>12 exp *"diffusion of innovation"/ (0)<br>13 *Information Dissemination/mt [Methods] (0)<br>14 *Program Evaluation/ (0)<br>15 *"Costs and Cost Analysis"/ (0)<br>16 9 or 10 or 11 or 12 or 13 or 14 or 15 (3035)<br>17 16 and 8 (50)<br>18 limit 17 to (english language and yr="2005 -Current") (43)<br>19 limit 18 to (evaluation studies or meta analysis or "review" or systematic reviews) (17)	17
Database: Embase <1996 to 2014 Week 33>	1 Health Services Research/ (21291)<br>2 ((health technolog$ adj3 assessment$) or hta).mp. (4277)<br>3 (NHS adj6 research$).mp. [mp=title, abstract, subject headings, heading word, drug trade name, original title, device manufacturer, drug manufacturer, device trade name, keyword] (584)<br>4 Biomedical technology assessment/ (9191)<br>5 Health Care Policy/ (119074)<br>6 *"medical research"/ (44171)<br>7 1 or 2 or 3 or 4 or 5 or 6 (192702)<br>8 (payback or pay back).ti,ab. (333)<br>9 (("benefit$" or "impact*" or "influenc$" or "gain$" or "utili#ation" or "returns") adj8 (research or evidence or health technolog$)).ti,ab. (68728)<br>10 ((implement$ or disseminat$) adj4 (benefit$ or impact* or gain$)).ti,ab. (4188)<br>11 program impact/ (73)<br>12 8 or 9 or 10 or 11 (72921)<br>13 7 and 12 (4868)<br>14 *"diffusion of innovation"/ (4800)<br>15 *"conceptual framework"/ (909)<br>16 *"empirical research"/ (455)<br>17 *"cost benefit analysis"/ (3972)	120

	18 *theoretical study/ (278)<br>19 post hoc analysis/ (9899)<br>20 *data analysis/ (2544)<br>21 *process model/ (493)<br>22 *practice guideline/ (35560)<br>23 program evaluation/ (1301)<br>24 *information dissemination/ (3309)<br>25 14 or 15 or 16 or 17 or 18 or 19 or 20 or 21 or 22 or 23 or 24 (63178)<br>26 13 and 25 (157)<br>27 limit 26 to (english language and yr="2005 -Current") (120)	
Cochrane Methodology Register Issue 3 of 4, July 2012	Impact AND research	61
Health Management Information Consortium (HMIC) 1979 to present Records from King's Fund and Department of Health (08/2014)	(impact AND "health research").ti,ab<br>Limited from 2005 to date	67
CINAHL	(impact AND "health research").ti,ab [Limit to: Publication Year 2005-2014]	188
Total records downloaded		947
Total records after duplicates removed and initial screening		297
